# Determinants of Herpes Zoster Vaccine Hesitancy: An Updated Systematic Review Based on the 5C Model

**DOI:** 10.3390/vaccines14100840

**Published:** 2026-09-23

**Authors:** Manuela Chiavarini, Anita Genga, Giorgia Maria Ricciotti, Pamela Barbadoro

**Affiliations:** 1 Department of Health Sciences, University of Florence, 50121 Firenze, Italy; manuela.chiavarini@unifi.it; 2 Department of Biomedical Sciences and Public Health, Section of Hygiene, Preventive Medicine and Public Health, Polytechnic University of the Marche Region, 60126 Ancona, Italy; p.barbadoro@staff.univpm.it

**Keywords:** herpes zoster, herpes zoster vaccine, vaccine hesitancy, vaccine willingness, 5C model, vaccination uptake, healthcare professional recommendation, adult immunization, vaccination determinants

## Abstract

**Background/Objectives:** Herpes zoster (HZ) represents a substantial public health burden, particularly among older adults and individuals at increased risk of severe disease. Despite effective vaccines, uptake remains suboptimal. This systematic review aimed to update evidence on HZ vaccination willingness, acceptance, and hesitancy, and to classify determinants according to the 5C model: Confidence, Complacency, Constraints, Calculation, and Collective Responsibility. **Methods:** A systematic literature search was conducted in PubMed, Web of Science, and Scopus from the date of the most recent relevant review (2022) to 15 June 2026. Eligible studies were observational studies assessing HZ vaccine hesitancy, willingness, acceptance, and related determinants among adults. Studies included in the previous systematic review were also reassessed. Study selection and data extraction were independently performed, and methodological quality was assessed using a modified Newcastle–Ottawa Scale. Due to substantial heterogeneity, findings were synthesized qualitatively. **Results:** Thirty-seven studies were included. Actual HZ vaccine uptake was generally low, despite moderate-to-high willingness in several populations. Healthcare professional (HCP) recommendation was the most consistent facilitator of vaccination. Within the 5C framework, low perceived susceptibility and severity of HZ, particularly its complications, were frequently associated with hesitancy, while vaccine cost, out-of-pocket payment, and limited access represented major constraints. Greater knowledge and awareness of HZ and HZ vaccination were generally associated with greater willingness, although knowledge alone did not consistently overcome hesitancy. Collective Responsibility was the least investigated domain. **Conclusions:** HZ vaccination uptake remains suboptimal despite favorable vaccination intentions. Strengthening HCP recommendations, reducing financial and structural barriers, and improving risk communication may enhance uptake. The 5C model provides a useful framework for understanding HZ vaccine hesitancy and informing targeted adult immunization strategies.

## 1. Introduction

### 1.1. Herpes Zoster Disease and Burden

Herpes zoster (HZ) results from reactivation of latent varicella zoster virus (VZV), with incidence increasing substantially with age and immunosenescence. Older adults and immunocompromised individuals are therefore at greatest risk. HZ represents a significant public health burden, with approximately one in three individuals developing the disease during their lifetime and an estimated 1.7 million new cases annually in Europe [1,2,3].

The burden of HZ extends beyond the acute episode, as complications such as postherpetic neuralgia (PHN) and ophthalmic and neurological involvement can result in persistent morbidity and impaired quality of life [4,5]. PHN is the most frequent chronic complication, occurring in approximately 5–20% of patients, with more than 30% experiencing pain persisting beyond 1 year [6]. HZ and PHN can substantially affect quality of life, sleep, daily functioning, mobility, social participation, and psychological well-being, while imposing considerable healthcare utilization and societal costs [7,8,9].

### 1.2. Herpes Zoster Vaccines

Given the substantial clinical, social, and economic burden of HZ, vaccination is the primary strategy for prevention, particularly in older adults and individuals at increased risk of severe disease [10]. Effective HZ vaccines are available and have been incorporated into national immunization programs for older and high-risk populations in many countries [11,12].

Two vaccines have been developed: the live attenuated zoster vaccine (ZVL; Zostavax^®^), licensed in 2006 [13], and the recombinant adjuvanted zoster vaccine (RZV; Shingrix^®^), licensed in 2017 [14]. Current international recommendations preferentially endorse RZV because of its superior and more durable efficacy and broader use in immunocompromised populations [15,16].

### 1.3. Herpes Zoster Vaccine Hesitancy

Despite the availability of safe and effective vaccines, vaccination uptake remains suboptimal in several settings, limiting the potential public health impact of vaccination [17]. In recent years, vaccine hesitancy has emerged as one of the major challenges to the success of immunization programs worldwide. The World Health Organization (WHO) Strategic Advisory Group of Experts (SAGE) initially defined vaccine hesitancy as a delay in acceptance or refusal of vaccination despite the availability of vaccination services [18], and it was included among the ten leading threats to global health in 2019 [19].

Vaccine hesitancy is recognized as a complex and context-specific phenomenon that varies across time, place, vaccines, and populations. Although vaccine hesitancy has been documented since the earliest immunization campaigns, it has emerged as a more complex public health challenge in the digital age, where social media facilitate the widespread and rapid dissemination of both information and misinformation, which can substantially influence vaccine-related attitudes and behaviors [20].

Understanding the factors influencing vaccination decisions has therefore become a critical priority for public health authorities, in order to develop targeted interventions aimed at improving HZ vaccine uptake.

Several theoretical frameworks have been proposed to explain vaccine-related behaviors, including the Health Belief Model (HBM) and vaccine-specific frameworks such as the 3C, 5C, and 7C models [18,21,22,23,24]. The HBM focuses primarily on individual perceptions of disease susceptibility and severity, perceived benefits and barriers, and cues to action [21,22]. Although useful for understanding individual health beliefs, it provides a relatively limited account of the broader psychological and social determinants of vaccination behavior.

The 5C model, developed by Betsch et al., extends the WHO’s 3C framework by identifying five psychological antecedents of vaccination: Confidence, Complacency, Constraints, Calculation, and Collective Responsibility [18,23]. These dimensions encompass not only perceptions of vaccine safety and disease risk, but also practical and psychological barriers, information-seeking and risk–benefit evaluation, and the willingness to protect others through vaccination. The model therefore integrates individual, interpersonal, and contextual determinants that are particularly relevant to vaccine uptake and to the design of targeted public health interventions [23,25].

The subsequent 7C model further expanded this framework by incorporating Compliance and Conspiracy [24].

While extensive research has focused on childhood immunization and, more recently, COVID-19 vaccination [26,27], hesitancy toward adult vaccines, including HZ vaccines, has received comparatively less attention despite its growing public health relevance. A recent systematic review and meta-analysis by Wang et al. synthesized global evidence on willingness to receive HZ vaccination and identified multiple associated factors across WHO regions [28]. However, the review primarily focused on estimating vaccine willingness, reporting that only approximately half of eligible individuals were willing to receive the HZ vaccine, and describing associated variables within the HBM framework.

Since the publication of Wang et al., additional studies investigating HZ vaccine acceptance, hesitancy, perceptions, and behavioral determinants have become available.

Moreover, given the growing application and established use of the 5C model in vaccine-hesitancy research, reclassifying and interpreting the emerging evidence on HZ vaccination through this lens may provide a broader characterization of the psychological and contextual mechanisms underlying vaccination decision [23,25].

An updated synthesis is therefore warranted to capture the evolving evidence base and to identify which dimensions of the 5C model are most consistently associated with HZ vaccine willingness across different populations and healthcare settings.

The present systematic review aims to update the evidence on HZ vaccine hesitancy and its determinants, previously examined using the Health Belief Model (HBM), by applying the WHO-derived 5C framework as an analytical model.

Specifically, we sought to (1) identify and synthesize studies evaluating HZ vaccine willingness, acceptance, or hesitancy; (2) classify reported determinants according to the five domains of Confidence, Complacency, Constraints, Calculation, and Collective Responsibility; and (3) examine how these psychological antecedents contribute to HZ vaccination decisions across different populations.

By synthesizing contemporary evidence through the WHO-derived 5C framework, this review seeks to enhance understanding of psychological, behavioral, and social determinants of HZ vaccination decisions. This multidimensional perspective may inform the development of tailored communication strategies and targeted interventions to address specific barriers to vaccination and contribute to strengthening adult immunization programs worldwide.

## 2. Materials and Methods

### 2.1. Protocol Registration

The Preferred Reporting Items for Systematic Review and Meta-Analyses 2020 (PRISMA 2020) guidelines [29] were followed when conducting the systematic review, and it was registered in the International Prospective Register of Systematic Reviews (https://www.crd.york.ac.uk/PROSPERO/ (accessed 24 February 2025), registration No: 2025 CRD420250651472).

### 2.2. Search Strategy and Data Sources

The search strategy was developed as an update to the systematic review and meta-analysis by Wang et al. [28], whose search covered studies published up to 20 June 2022.

A systematic literature search was conducted in PubMed (http://www.ncbi.nlm.nih.gov/pubmed/), Web of Science (http://wokinfo.com/), and Scopus (https://www.scopus.com/) from the date of the most recent relevant review (2022) to 15 June 2026 to identify original studies investigating willingness to vaccinate against HZ and its associated factors.

Accordingly, all studies included in the previous review were screened for eligibility according to the inclusion and exclusion criteria of the present study, and were incorporated when appropriate.

Detailed search strategies, including keywords and Boolean operators, are reported in Table S1. Only articles published in English were considered eligible.

To ensure comprehensive retrieval of the evidence, the reference lists of relevant reviews and eligible studies were also screened (backward citation searching) to identify additional relevant articles published before 2022.

All records retrieved from the database searches and reference screening were imported into Rayyan, a web-based application for systematic reviews, for deduplication, management, and study selection [30].

### 2.3. Inclusion and Exclusion Criteria

Studies were included if they met the following inclusion criteria:

**Study Design**
  Observational studies, including cross-sectional studies, were eligible.

**Population**
  Adults aged 18 years and older.

**Exposure/determinants**
  Psychological, informational, socioeconomic, healthcare-related, and structural determinants of HZ vaccination hesitancy/willingness.

**Comparator**
  Not applicable/reference category defined within individual studies.

**Outcomes**

- Primary: HZ vaccine willingness, acceptance, hesitancy, and uptake.
- Secondary: Determinants associated with these outcomes.

Studies were excluded if they were:

- Non-observational or non-cross-sectional;
- Focused on populations under 18 years;
- Not reporting outcomes related to HZ vaccine willingness, acceptance, or hesitancy;
- Not published in English.

Gray literature was not systematically searched because the review focused on peer-reviewed observational studies. Observational studies were selected because the objective of this review was to investigate population-level determinants, perceptions, willingness, and hesitancy toward HZ vaccination in real-world settings rather than to estimate the effectiveness of an intervention.

### 2.4. Study Selection

After removing duplicate records, three researchers independently screened articles first by title and abstract and then on the basis of full texts. Disagreements were resolved through discussion among the review team members. Reviews and meta-analyses were excluded. No studies were excluded for weaknesses in design or data quality.

### 2.5. Data Extraction

Two investigators independently extracted data using a standardized form capturing the following: title, first author, journal, publication year, study location, study design, sample size, participant characteristics (age, sex, race/ethnicity, education level), disease and socio-cultural and economic context, vaccines assessed, study outcomes (including vaccination willingness rates), preparatory materials provided, and factors influencing vaccine hesitancy. A third investigator verified data accuracy, and any disagreements were resolved through consensus among all authors.

According to data extraction, articles explored different aspects of HZ acceptance and hesitancy, adopted different tools for investigation (e.g., questionnaires, interviews, etc.), and were performed in various contexts. Because of all these reasons and the heterogeneity of the information collected, data synthesis was conducted only qualitatively.

### 2.6. Risk-of-Bias Quality Assessment

The methodological quality of the included cross-sectional studies was assessed using a modified version of the Newcastle–Ottawa Scale (NOS) adapted for cross-sectional designs [31], with a maximum score of 10 points. The scale evaluates three domains:

- Selection (maximum 5 points): Representativeness of the sample, sample size, non-respondents, and ascertainment of exposure/outcome.
- Comparability (maximum 2 points): Control for potential confounding factors.
- Outcome (maximum 3 points): Outcome assessment and statistical analysis.

Scores were interpreted as follows:

- 8–10 points = High quality;
- 6–7 points = Moderate quality;
- ≤5 points = Low quality.

A summary of the NOS-modified assessment for each study is presented in the last column of Table 1, while the detailed NOS evaluation for each individual study is provided in the Supplementary Material.

Two investigators independently carried out the selection of studies, extracted the data, and assessed the quality of the research. Any disagreements were settled through discussion or by involving a third author.

The risk-of-bias (RoB) of the included observational studies was assessed independently by two reviewers using a modified Newcastle–Ottawa Scale (NOS) adapted for cross-sectional studies, with scores ranging from 0 to 10. Studies scoring 8–10, 6–7, and ≤5 were classified as having a low, moderate, and high risk-of-bias, respectively. Any disagreements between reviewers were resolved through discussion and consensus, with consultation with a third reviewer when necessary.

Given the absence of a quantitative meta-analysis, statistical methods for assessing small-study effects or publication bias, such as funnel plots or formal statistical tests, were not applied. Potential reporting and publication biases were instead considered qualitatively when interpreting the findings.

### 2.7. Statistical Analysis and Data Synthesis

Descriptive statistics were used to summarize the characteristics of the included studies, including publication year, country, study design, sample size, population characteristics, vaccination uptake, and willingness, hesitancy, or acceptability estimates, when reported. For categorical variables, findings were summarized using frequencies and percentages; continuous variables were described using the summary measures reported by the individual studies (e.g., means, medians, ranges, or standard deviations).

Substantial heterogeneity was observed across the included studies in several key domains, including participant populations (e.g., different age groups, different health status), study settings, outcome definitions (e.g., HZ vaccination willingness, hesitancy, and acceptability), measurement instruments used to assess these outcomes, and analytical approaches, including the use of different predictors, adjustment strategies, and reported effect measures. These differences also persisted across potentially comparable subsets of studies, such that a sufficiently consistent outcome definition and common effect measure could not be established a priori for quantitative pooling. Therefore, meta-analysis and pooled effect estimates were considered inappropriate, as quantitative pooling could have obscured clinically and methodologically important differences between studies.

Findings were consequently synthesized narratively and organized according to the five domains of the 5C framework (Confidence, Complacency, Constraints, Calculation, and Collective Responsibility). Associations reported by individual studies were considered in the qualitative synthesis according to their direction and consistency across studies; statistical significance was not used as the sole criterion for determining the importance of a determinant.

Because not all included studies were originally designed according to the 5C framework, reported determinants were mapped to the five 5C domains using predefined conceptual criteria based on the primary construct assessed by each variable. Determinants reflecting trust in vaccination and perceptions of vaccine safety, efficacy, effectiveness, or reliability were classified as Confidence. Those concerning perceived susceptibility to infection, perceived severity of the disease, or perceived need for vaccination were classified as Complacency. Practical, structural, or logistical barriers and facilitators to vaccination, including access, availability, convenience, and cost, were classified as Constraints. Determinants concerning the acquisition, evaluation, or weighing of information, including perceived benefits and risks of vaccination and risk–benefit considerations, were classified as Calculation. Those related to responsibility towards others and contribution to the protection of one’s family or community were classified as Collective Responsibility.

When a determinant could reasonably correspond to more than one domain, it was assigned according to its primary conceptual meaning and the specific construct assessed by the original study, based on the wording and context in which the variable was reported. This approach was applied consistently across studies.

## 3. Results

The literature search revealed 220 studies from the PubMed database, 324 from Web of Science, and 373 from Scopus. After removing duplicates (*n* = 401), we identified 516 records screened for title and abstract revision (Figure 1).

Among these, 474 records were excluded because they did not meet the predefined eligibility criteria. The full texts of 42 articles were assessed for eligibility. All reports were successfully retrieved. Following full-text evaluation, 12 studies were excluded for the following reasons: qualitative study design (*n* = 5), assessment of vaccine uptake without evaluation of vaccine hesitancy (*n* = 3), review articles (*n* = 2), and studies addressing vaccine hesitancy in general rather than focusing on the hesitancy of HZ vaccination (*n* = 2).

A total of 30 studies met the inclusion criteria and were included in the systematic review. Additionally, among 10 studies found in the Wang review, only seven studies were included in this review, because they were eligible according to our inclusion criteria [28]. Ultimately, 37 total studies were included in our systematic review [32,33,34,35,36,37,38,39,40,41,42,43,44,45,46,47,48,49,50,51,52,53,54,55,56,57,58,59,60,61,62,63,64,65,66,67,68].

Overall, all 37 included studies were assessed as having NOS scores ≥7. Globally, 33 studies were assessed as having a low risk-of-bias and a NOS score between 8 and 10, while only four studies were assessed as having a moderate risk-of-bias and a NOS score of 7 (Table 1). However, potential limitations related to representativeness, outcome measurement, confounding, and cross-sectional design were considered when interpreting the findings.

**Table 1:** General characteristics of the 37 included studies [32,33,34,35,36,37,38,39,40,41,42,43,44,45,46,47,48,49,50,51,52,53,54,55,56,57,58,59,60,61,62,63,64,65,66,67,68].

First Author, Year, Country, [Ref]	Characteristics of the Study Population (N), Gender (F%), Age (y), Chronic Diseases	Setting, HCP Recommendation (Yes/No/%), HZV Uptake (%)	Endpoint (Hesitancy, Willingness, Acceptability)	NOS	RoB
Peng 2026 China [32]	N: 420; F: 60.7% Age ≥ 60, mean 68.3 Poor health status: 7.6%	Community; cross-sectional survey - -	Willingness: 16.7%	8	Low
Deng 2026 China [33]	N: 1974; F: 54.0% Age ≥ 60, mean 67.3 Chronic diseases: 56.4%	Community; cross-sectional survey through healthcare centers 17.7% received HCP recommendation 8.4% received HZV	Willingness: 33.7% Hesitancy: 27.8%	9	Low
Lim 2026 Philippines [34]	N: 402; F: 63.2% Age ≥ 50, mean 63.0 ± 8.03 Hypertension: 62.7%, DM: 23.4%	Community, outpatient clinic; cross-sectional survey - -	Willingness: 87.0% Willingness if advised by HCP: 96.9%	7	Moderate
Alrufayyiq 2026 Saudi Arabia [35]	N: 386; F: 58.6% Age ≥ 50; 50–55: 76.2%; 56–50: 15%; ≥61: 8.8% Chronic diseases: 46.6%	Community; cross-sectional offline and online survey - -	Willingness if advised by HCP: 60.1%	8	Low
Alolayan 2026 Saudi Arabia [36]	N: 439; F: 53.1% Age ≥ 50; mean 56.9; 50–64: 87.2%; ≥65: 12.8% Chronic diseases: 77%	Community; cross-sectional survey at primary healthcare centers 22.8% received HCP recommendation 10.5% received HZV	Willingness: 29.5%	8	Low
Yang 2026 China [37]	N: 1184; F: 54.6% Age ≤ 40: 24.0%; 40–49: 31.8%; 50–59: 30.7%, ≥60: 13.5% Chronic diseases: 10.6%	Community; online and offline survey - -	Vaccination intention (Scale)	9	Low
Qu 2026 China [38]	N: 964; F: 57.1% Age ≥ 50; 50–59: 49.5%, ≥60: 50.5% Chronic diseases: 74.4%	Community; multicenter cross-sectional survey - -	HZV intention (Scale)	10	Low
Dai 2026 China [39]	N: 12,357 Age ≥ 60 y -	Community; cross-sectional survey in community health centers 17.6% received HCP recommendation (in highest profile) -	“Willing but Obstructed” profile: 44.6%	10	Low
Zhang 2026 China [40]	N: 772; F: 53.0% Age ≥ 60; mean 68.0 ± 6.67 Chronic diseases: 100%	Community; multicenter cross-sectional survey - 11.7% received HZV	Willingness: 26.7%	9	Low
Liu 2026 China [41]	N: 2047; F: 63.8% Mean age: 46.8 (range 18–59) History of HZ: 12.9%	Community; cross-sectional survey - -	Willingness: 10.3%	9	Low
Oku 2025 Japan [42]	N: 1637; F: 82.9% Mean age: 58.6 AIRD patients	Hospital/clinics; cross-sectional survey 9.3% received HCP recommendation 8.8% received HZV	Willingness: 22.2%	8	Low
Hou 2025 China [43]	N: 3007; F: 53.6% Age ≥ 50; 50–59: 67.6%; ≥60: 32.3% Chronic diseases: 42%	Community; cross-sectional offline and online survey 39.8% received information from HCP -	Hesitancy: 62.7–68.9% across regions	9	Low
AlSaif 2025 Saudi Arabia [44]	N: 333; F: 47.4% Age ≥ 50; mean 60.4 ± 7.6 Chronic diseases: 83.5%	Community, outpatient clinics; cross-sectional interview 9.6% received HCP recommendation 12% received HZV	Willingness: 45.7%	9	Low
Zhu 2025 China (Ningbo) [45]	N: 6202; F: 49.24% Age ≥ 50; mean 67.1; 50–59: 25.5%; 60–69: 30.0%; ≥70: 44.4% Chronic diseases: 71.94%	Community; population-based cross-sectional survey (online + face-to-face) 39.18% received HCP recommendation -	Willingness: 36.05%	9	Low
Gao 2025 China (Shanghai) [46]	N: 1492; F: 64.2% Age ≥ 40; median 53 Chronic diseases: 51.6%	Community; cross-sectional survey (online questionnaire) - -	Hesitancy: 80.5%	9	Low
Liang 2025 Hong Kong [47]	N: 673; F%: 55.8% Age ≥ 65; 65–69: 66.7%; 70–74: 21.2%; ≥75: 12.1% Chronic diseases: 54.4%	Community; random telephone cross-sectional survey - -	Willingness (self-financed): 7.7% Willingness (free vaccine): 37.3%	9	Low
Motet 2025 Belgium [48]	N: 327; F: 26.9% Age: 49 ± 12.2 HIV patients Chronic diseases other than HIV: 23.8%	HIV outpatient clinic; cross-sectional survey (self-administered questionnaire) - -	Willingness: 61.7%	8	Low
Wang 2025 China (Ningbo) [49]	N: 2025; F%: 59.9% Age ≥ 40; mean 59.8 ± 12.2 40–49: 25.5%; 50–59: 26.9%; ≥60: 47.6% Chronic diseases: 37.6%	Community; cross-sectional survey (face-to-face questionnaire, multistage sampling) - -	Hesitancy	9	Low
Chang 2024 China [50]	N: 1246; F: 60.6% Age ≥ 40; mean 56.2 ± 9.6 40–49: 27.4%; 50–59: 36.0%; 60–69: 28.0%; 70–79: 7.7%; ≥80: 1.0% Chronic diseases: various	Community + hospitals; cross-sectional survey (electronic questionnaire, face-to-face assisted) Yes -	Willingness: 87.1% Willingness if advised by HCP: 78.9%	9	Low
Dyda 2024 Australia [51]	N: 1012; F: 52.2% Age ≥ 50; mean 66.4 ± 10.8 (range 50–94) -	Community; national cross-sectional survey (online + telephone; general population) Yes -	Acceptance: 67.4% consider vaccination important (proxy for acceptance) Hesitancy: 32.6%	9	Low
Lasagna 2024 Italy [52]	N: 309; F: 50.5% Age range 59–76; median: 67 Cancer patients	Oncology outpatient clinic; cross-sectional offline survey 37% received HCP recommendation 32.4% received HZV	Acceptability	7	Moderate
Salussolia 2024 Italy [53]	N: 1810; F: 43.4% Age ≥ 50; 50–64: 23.6%; ≥65: 76.4% Chronic diseases: DM 24.4%, cardiovascular diseases 19.0%, respiratory diseases 12.7%	Community; nationwide cross-sectional survey (CAWI—online panel; stratified sample) -	Hesitancy	10	Low
Rubaian 2024 Saudi Arabia [54]	N: 8138; F: 51.8% Age < 50: 72.8%; 50–64: 24.1%; ≥65: 3.1% Chronic diseases: diabetes 16%, hypertension 13.6%	Community; cross-sectional survey (online questionnaire via social media) 12.3% received HCP recommendation -	Willingness: 24% Neutral: 34.1%	7	Moderate
Zhang 2024 China [55]	N: 3040; F: 62.63% Age ≥ 50; median 66 (range 50–91) 50–59: 53.22%; 60–69: 30.92%; 70–79: 13.06%; ≥80: 2.80% Chronic diseases: 38.75% (e.g., hypertension 26.15%, hyperlipidemia 16.94%, DM 12.11%)	Community; cross-sectional survey (online questionnaire, multistage sampling) 39.57% received HCP recommendation 13.26% received HZV	Willingness: 52.43% Unwilling: 34.31%	9	Low
Grudziaz-Sekowska 2024 Poland [56]	N: 1137; F: 55.6% Age range 18–88; ≥50: 46.5% Chronic diseases: 22.3%	Community; cross-sectional survey via e-mail and text message 30.5% received HCP recommendation -	Willingness: 63.7%	9	Low
Wang 2023 China [57]	N: 2864; F: 61% Age range 25–95; 40–59: 32.4%; ≥60: 28.7% Chronic diseases: 22.4%	Community; cross-sectional survey via e-mail and text message - -	Willingness: 42.7%	9	Low
Di Giuseppe 2023 Italy [58]	N: 515; F: 52% Age ≥ 50; ≥65: 69.1% Chronic diseases: 30.3%	Community, GP clinic; interview 4.9% received information from HCP -	Willingness: 26.6%	9	Low
Maertzdorf 2023 Netherlands [59]	N: 2933; F: 52.9% Age range 46–86; ≥65: 46%; 65–75: 32.8%; ≥75: 13.2% Chronic diseases: 57.5%	Community; cross-sectional data from the population-based Doetinchem Cohort Study - -	Willingness: 23%	9	Low
Al-Orini 2023 Saudi Arabia [60]	N: 410; F: 34% Age range 53–62 DM patients	Primary healthcare center; cross-sectional study with online questionnaire - -	Acceptability: 25.4% Acceptability if advised by HCP: 74.2%	8	Low
Jiang 2022 China [61]	N: 3838; F: 49.4% Age ≥ 50; 50–59: 65.7%; 60–69: 21.1%; ≥70: 13.2% Chronic diseases: 34.9%	Community; online questionnaire survey 28.4% received HCP recommendation 10.34% received ≥1 dose of HZV	Willingness: 43%	9	Low
Al-Khalidi 2022 United Arab Emirates [62]	N: 420; F: 45.2% Age ≥ 50; 50–55: 61%; 56–60: 23.1%; >61: 16% Chronic diseases: 66%	Community; cross-sectional study: in-person public venues questionnaire - -	Willingness: 71.9% Willingness if advised by HCP: 83.6%	8	Low
Lu 2021 China [63]	N: 1672; F: 53.5% Age ≥ 50 (range 50–69); 59–59: 43.5%; 60–69: 56.5% -	Community; face-to-face questionnaire - -	Willingness to get HZV by self-payment: 16.57% Willingness if covered under insurance: 72.25%	8	Low
Nicholls 2021 UK [64]	N: 372; F: 49.7% Age ≥ 65 (range 65–92); mean 70.5 -	Community; online cross-sectional survey 63.2% received HCP recommendation 58.9% received HZV	Willingness: 34.6%	8	Low
Del Signore 2020 France [65]	N: 907; F: 55.3% Age ≥ 65; mean: 75.7 ± 7.4 Chronic diseases: 36.7%	Hospital and outpatient clinic; face-to-face interview - -	Willingness if eligible/risk factors: 68.9%	8	Low
Baalbaki 2019 USA [66]	N: 373; F: 65% Age ≥ 50; <60: 29.5%; ≥60: 70.5% -	Community; face-to-face interview 19.1% in <60 y group received HCP recommendation 43.3% in ≥60 y group received HCP recommendation -	Willingness in <60 y group: 46.4% Willingness in ≥60 y group: 50.2%	7	Moderate
Yang 2015 South Korea [67]	N: 603; F: 55.1% Age 19–49: 40.5%; ≥50: 59.5% Chronic diseases: 26.5%	Hospital, outpatient clinic; cross-sectional survey 22% of ≥50 y received HCP recommendation 3.1% of ≥50 y received HZV	Willingness: 85.8% Willingness if advised by HCP: 86.9% Willingness considering the present cost: 59.6%	9	Low
Lu 2009 USA [68]	N: 3662; F: 54.6% Age ≥ 60; 60–74: 66.5%; ≥75: 33.5% -	Community; telephone survey - 1.9% received HZV	Willingness if advised by HCP: 77.8%	9	Low

HCP: Healthcare Professional, HZ: Herpes Zoster, HZV: Herpes Zoster Vaccine, -: Not reported, AIRD: Autoimmune Inflammatory Rheumatic Disease, DM: Diabetes Mellitus.

Overall, the 37 included studies were predominantly cross-sectional in design, most frequently administered as online, face-to-face, or telephone questionnaires within community settings; a smaller number recruited participants from hospital or outpatient clinic settings, including oncology [52], HIV [48], rheumatology [42], and diabetes [60] clinics, while one study drew on longitudinal cohort data [59]. Geographically, studies were conducted across four continents, with the largest contribution from China (*n* = 16) [32,33,37,38,39,40,41,43,45,46,49,50,55,57,61,63], followed by Saudi Arabia (*n* = 5) [35,36,44,54,60], Italy (*n* = 3) [52,53,58], and single studies from the Philippines, Japan, Australia, the Netherlands, Poland, the United Arab Emirates, the United Kingdom, France, Belgium, South Korea, and Hong Kong [34,42,47,48,51,56,59,62,64,65,67], and two from the United States [66,68].

Sample sizes ranged from just over 300 [52] to more than 12,000 participants [39], and study populations were mostly composed of adults aged 50 years and older, although a few studies included adults from 18 [41,56] or 40 [46,49,50] years of age. Where reported, chronic disease prevalence among participants varied widely, reflecting the inclusion of both general-population samples and clinical populations selected for underlying conditions such as cancer [52], HIV [48], rheumatic disease [42], and diabetes [60].

Reported vaccination-related outcomes were heterogeneous and were distinguished according to whether they reflected attitudes toward vaccination, vaccination intentions, or actual vaccination behavior. Willingness, hesitancy, and acceptability were considered attitudinal outcomes, while structured vaccination-intention scales were considered intention-related outcomes. Actual HZV uptake, when assessed, was considered a behavioral outcome. Willingness was the most frequently reported endpoint, whereas vaccination intention and actual vaccine uptake were assessed in a smaller number of studies. Across the studies reporting actual uptake, HZV coverage was consistently low, generally below 15% [33,36,40,55,61,67,68]. The proportion of participants who had received a HCP recommendation for vaccination was also low [33,36,43,44,45,54,55,58,61,67], although recommendation from a HCP was consistently associated with higher willingness, acceptability, vaccination intention, or actual uptake, depending on the outcome assessed [34,42,44,50,60,62,63,64,66].

The 37 included studies explored a wide range of influencing factors across the five dimensions of the 5C model, namely Confidence, Complacency, Constraints, Calculation, and Collective Responsibility (Table 2).

Table 3 summarizes, for each of the 37 included studies, the factors found to be associated with HZ vaccine willingness, hesitancy, or acceptability, organized according to socio-demographic determinants and the five domains of the 5C model described in Table 2.

Although 37 studies were included, the number of studies alone was not considered sufficient to justify quantitative pooling. A meta-analysis requires sufficient clinical, methodological, and statistical comparability among studies. In the present review, substantial heterogeneity was observed in the populations studied (general populations and selected clinical groups), age distributions, geographical and healthcare contexts, study settings, and vaccination policies. In addition, the reported outcomes were not uniform and included willingness, hesitancy, acceptability, vaccination intention, and, in a subset of studies, actual vaccine uptake. These outcomes were measured using different questionnaires, scales, response categories, hypothetical vaccination scenarios, and definitions of willingness or hesitancy. Furthermore, the determinants investigated and the statistical measures used to describe their associations varied substantially across studies. Therefore, combining these estimates would risk producing a statistically precise but conceptually misleading pooled estimate. A quantitative synthesis was consequently considered inappropriate, and a structured narrative synthesis was judged to provide a more valid representation of the available evidence.

The determinants identified in the included studies should be interpreted in relation to the specific vaccination-related outcome assessed. Most studies investigated attitudinal or intention-related outcomes, particularly willingness, hesitancy, acceptability, and vaccination intention, whereas a smaller subset assessed actual HZV uptake. Accordingly, the following narrative synthesis primarily describes determinants of vaccination attitudes and intentions, while findings related to actual uptake are reported separately where available.

Socio-demographic determinants showed considerable heterogeneity across studies and populations. Age was among the most frequently examined variables, but its direction of association was inconsistent: some studies reported that older age was associated with greater willingness or acceptability [33,51,52,58,59,66], while an equally large number reported the opposite, with older participants showing lower willingness or greater hesitancy [45,46,47,49,57,63,67]. A similar pattern of inconsistency was observed for gender, educational level, and the presence of chronic disease, which were positively associated with the outcome in some studies [33,58,59,60,63,65,67], negatively associated in others [43,45,53,55,61], and unrelated in a substantial proportion of studies [35,40,44,56,68]. Income and insurance status showed a more consistent pattern, being repeatedly linked to structural, cost-related barriers to vaccination rather than to willingness per se [39,45,49,50,51,53,55,63].

Within the Confidence domain, the single most consistent determinant across the included studies was a recommendation from a healthcare professional (HCP), which was associated with substantially higher willingness, acceptability, or uptake in nearly every study that assessed it [33,34,35,42,44,50,51,54,55,60,62,66]. Conversely, low trust in vaccine safety or effectiveness, and the absence of a HCP recommendation, were consistently associated with greater hesitancy [39,43,49,53]. Perceived response efficacy of the vaccine and a positive attitude toward HZ vaccination were also generally positively associated with willingness [32,34,38,44,57,58,65].

Within the Complacency domain, low perceived susceptibility to HZ and, especially, low perceived severity of the disease or its complications, were the most frequently and consistently reported barriers to vaccination, being associated with lower willingness or higher hesitancy across the majority of studies that examined them [38,43,44,49,50,51,52,53,54]. A personal history of HZ, or knowing someone affected by the disease, tended to increase perceived risk and, correspondingly, willingness to vaccinate [34,36,46,63], although this association was not universal [49,65,67].

Within the Constraints domain, the cost of vaccination and, more broadly, structural or financial barriers, including lack of insurance coverage and out-of-pocket payment, emerged as some of the most consistently reported obstacles to HZ vaccination across studies conducted in both high- and middle-income settings [32,33,34,39,45,46,49,50,51,53,55]. Limited access to vaccination services, particularly in rural or under-resourced areas, was also repeatedly identified as a barrier [49,53,61].

Within the Calculation domain, knowledge and awareness of HZ and the HZ vaccine were the most frequently examined determinants and were generally positively associated with willingness [33,42,44,45,47,54,55,57,63], although several studies found no significant association [34,60,65], suggesting that knowledge alone is not always sufficient to overcome hesitancy. Concerns about vaccine side effects and reliance on informal information sources, such as family, friends, or social media, were more consistently associated with greater hesitancy [40,47,50,52,54].

Finally, the Collective Responsibility domain was the least frequently assessed dimension across the included studies, with the large majority reporting no data on this construct. Among the few studies that did report on it, a stronger sense of responsibility toward vulnerable populations, such as family members or the wider community, was consistently associated with greater willingness [37,39,49,64].

## 4. Discussion

This study should be interpreted as an updated systematic review rather than a replication of Wang et al. [28]. Their 2023 systematic review and meta-analysis included studies available up to 20 June 2022, and primarily assessed willingness to receive HZ vaccination and associated factors using the Health Belief Model. The present review updates the evidence through 15 June 2026, retaining seven studies from the previous review and identifying 30 additional eligible studies, for a total of 37 studies.

The present review also differs conceptually and analytically from Wang et al. First, its primary objective is broader than estimating willingness alone, encompassing vaccine hesitancy, willingness, acceptance, and the determinants underlying vaccination decisions. Second, determinants are systematically classified according to the 5C framework—Confidence, Complacency, Constraints, Calculation, and Collective Responsibility—rather than being primarily interpreted through the Health Belief Model. Third, the present review incorporates more recent evidence published after mid-2022, including studies conducted in contemporary vaccination-policy and healthcare contexts. Finally, unlike the previous review, no pooled quantitative estimate is presented because the substantially expanded evidence base is also characterized by marked heterogeneity in populations, outcome definitions, measurement tools, and study methods. Thus, the added value of the present review lies not only in updating the number of available studies but also in providing a contemporary, theoretically structured interpretation of the determinants of HZ vaccine hesitancy.

Because the included studies assessed several conceptually distinct outcomes—willingness, hesitancy, acceptability, vaccination intention, and, less frequently, actual vaccine uptake—these are not inter-changeable, and were therefore considered separately when interpreting the evidence. Willingness, intention, and acceptability are attitudinal con-structs measured at a single time point and are, by definition, upstream of behavior; hesitancy captures ambivalence or reluctance that may or may not translate into a decision, and only actual uptake reflects realized vaccination behavior. Determinants identified within the 5C framework predominantly explain variation in the attitudinal outcomes, whereas the much smaller subset of studies reporting uptake data points to a distinct set of structural and system-level barriers (cost, accessibility, recommendation pathways) that operate downstream of intention. This distinction is essential to avoid conflating predictors of stated willingness with predictors of actual vaccination, and is carried through the remainder of this section.

This framework provides a complementary and more behaviorally oriented interpretation of the evidence, distinguishing psychological antecedents such as confidence and complacency from structural and practical constraints, information-seeking and risk–benefit calculation, and collective responsibility.

This broader behavioral perspective is consistent with recent theoretical work showing that vaccination decisions may emerge from the interplay between individual decision-making, social interactions, and the wider dynamics of infectious disease transmission [69].

The present review therefore does not simply reproduce the previous evidence synthesis but updates it while addressing a different analytical question.

This study highlights substantial variability in attitudes towards HZ vaccination across different geographical and healthcare settings. Among studies reporting a directly quantifiable measure of willingness to receive HZ vaccination, willingness ranged from 7.7% to 87.1%, with a descriptive mean of 43.8% and a median of 42.7%.

These figures should be interpreted as descriptive summaries of the reported esti-mates rather than as a pooled quantitative measure, given the substantial heterogeneity in study populations, definitions, vaccination costs and accessibility, healthcare settings, and measurement approaches. Overall, willingness was moderate-to-high in several settings, whereas vaccine hesitancy was also frequently reported, with estimates ranging from approximately one-third of participants to more than 80% in some populations. This contrast between willingness and hesitancy suggests that intention to vaccinate is strong-ly influenced by contextual factors, including the perceived susceptibility and severity of HZ, confidence in vaccine safety and effectiveness, cost, access, and recommendation from a healthcare professional.

Marked geographical differences were also observed. In China, where 16 of the in-cluded studies were conducted, willingness estimates ranged from 10.3% to 52.4% when a directly reported percentage was available, while several studies assessed vaccination intention using scales or identified distinct attitudinal profiles rather than a single percent-age estimate. Hesitancy was particularly pronounced in some Chinese settings, reaching 62.7–68.9% across regions and 80.5% in one Shanghai-based study. These findings should therefore not be interpreted simply as evidence of uniformly low acceptance, but rather as reflecting considerable heterogeneity within the Chinese context itself. In other Asian settings, willingness was substantially higher in some studies, including 87.0% in the Phil-ippines and 85.8% in South Korea, although willingness was lower when out-of-pocket costs were considered. Similar variability was observed in Middle Eastern, European, and Western countries, underscoring the influence of local policy, vaccine availability, financing mechanisms, healthcare delivery models, and professional recommendation on HZ vaccination attitudes.

Notably, actual vaccination uptake—reported in a minority of the included studies—remained consistently lower than stated willingness across virtually all settings, gener-ally below 15%. This gap between attitudinal outcomes and realized behavior indicates that improving willingness alone is unlikely to be sufficient to increase population-level uptake, and it reframes the determinants discussed below as operating at different points along the pathway from attitude to vaccination.

Applying the 5C framework, Confidence—and, in particular, recommendation from a healthcare professional (HCP)—and Constraints, especially vaccine cost and structural access barriers, emerged as the most consistent determinants of vaccination behavior across populations. Low perceived susceptibility and severity within the Complacency domain were also frequently reported barriers, whereas Collective Responsibility remained the least investigated dimension, despite the studies that did assess it consistently linking it to greater willingness.

These findings extend the 2023 systematic review and meta-analysis by Wang et al. [28], which relied on the Health Belief Model and covered evidence up to mid-2022, by incorporating more recent studies and reframing determinants through the 5C lens.

Beyond reorganizing known barriers, the 5C framework provides added interpretive value by situating HZ vaccine hesitancy within the broader vaccine-hesitancy literature and by linking determinants to actionable intervention targets. Unlike the HBM, its domains facilitate a more differentiated approach, suggesting that interventions should be tailored to the predominant barriers rather than rely on generic awareness strategies.

The substantial intention–behavior gap indicates that willingness alone may not translate into vaccination. Findings can be conceptualized across three interacting levels: individual factors shaping willingness or hesitancy; HCP factors, particularly recommendation and risk communication; and health-system factors, including cost, reimbursement, accessibility, availability, and vaccination pathways. This supports the need for multilevel interventions addressing both attitudes and structural barriers.

The review did not systematically assess reimbursement or healthcare-access contexts, although willingness appeared lower where out-of-pocket costs were reported. Future studies should therefore report these contextual factors alongside attitudinal outcomes. Similarly, few studies specified the HZ vaccine considered, limiting assessment of vaccine-specific effects on hesitancy and highlighting the need to incorporate product-specific efficacy, safety, reactogenicity, and cost in future research.

This approach offers a more granular and psychologically grounded review of barriers and facilitators, consistent with the growing adoption of the 5C model in the broader vaccine-hesitancy literature, and may better support the design of targeted interventions than earlier disease-specific frameworks.

### 4.1. Strengths and Limitations

This review has several strengths: a prospectively registered protocol, a systematic search across three major databases complemented by reference-list screening, adherence to PRISMA 2020 methodology (Table S3), independent duplicate screening and data extraction with third-reviewer adjudication, and formal quality appraisal of included studies using a modified Newcastle–Ottawa Scale. The use of a well-established theoretical framework (5C) additionally allowed heterogeneous findings to be classified within a common, clinically interpretable structure.

Several limitations should be acknowledged. First, because of substantial heterogeneity in populations, outcome definitions (willingness, hesitancy, acceptability, intention), and measurement tools, data synthesis was necessarily qualitative, precluding pooled quantitative estimates. Second, included studies were geographically skewed, with China and Saudi Arabia together contributing over half of the evidence base, limiting the generalizability of findings to other WHO regions, particularly low-resource settings. Third, nearly all included studies were cross-sectional, preventing causal inference regarding the determinants identified.

Fourth, restricting eligibility to English-language publications may have excluded relevant studies from non-English-speaking contexts and cannot exclude publication bias, particularly because gray literature was not systematically searched.

Finally, the Collective Responsibility domain was assessed in only a minority of studies, limiting the strength of conclusions regarding its role in HZ vaccination decisions.

From a public health perspective, recommendation from a HCP stands out as the single most consistent and modifiable driver of HZ vaccine uptake, supporting the integration of vaccination prompts or standing orders into routine primary and specialist care, particularly for older adults and immunocompromised patients. Addressing low perceived severity, especially regarding postherpetic neuralgia and other complications, and reducing out-of-pocket cost through funding or subsidy schemes are likely to yield meaningful gains in willingness and uptake.

Although Collective Responsibility was investigated in only a small number of studies, the available evidence consistently suggested a positive association with vaccination willingness, so—while this finding should be considered hypothesis-generating rather than conclusive—framing HZ vaccination in terms of collective and familial protection may represent a promising, low-cost communication strategy for future adult immunization campaigns.

### 4.2. Implications for Practice

The findings of this study translate into concrete implications across several levels of the vaccination decision-making process.

At the individual level, interventions should aim to improve risk perception of the target disease, providing clear information on potential complications in at-risk groups. Educational messaging should also directly address the safety concerns most frequently reported by respondents, as unresolved safety-related doubts emerged as a key barrier to vaccine uptake.

At the HCP level, our results confirm that recommendation by a healthcare professional was among the most consistent determinants of vaccination uptake. This underscores the need for a systematic recommendation strategy, in which vaccination is proposed by HCPs rather than left to patient initiative. Embedding vaccination prompts within electronic health records or routine clinical workflows could support this systematic approach, while opportunistic vaccination during unrelated clinical visits (e.g., chronic disease follow-up, hospital admissions) may further reduce missed opportunities.

At the system level, given that cost and accessibility were identified as significant barriers, policies aimed at reducing out-of-pocket costs and improving accessibility (e.g., extended clinic hours, vaccination points outside traditional healthcare settings, pharmacy-based administration) are likely to increase uptake, particularly among socioeconomically disadvantaged groups. Integrating vaccination into routine care pathways, rather than treating it as a separate preventive act, may further reduce structural barriers to access.

Finally, in terms of communication, our findings support the need for tailored communication strategies rather than generic public health messaging. Communication should be adapted to the specific concerns, risk perception, and informational needs identified within each subgroup, in order to maximize its persuasive and educational impact.

## 5. Conclusions

HZ vaccine uptake remains suboptimal worldwide despite generally favorable willingness, driven mainly by lack of a recommendation from a HCP and structural or cost-related barriers. The 5C model offers a structured framework for characterizing determinants of HZ vaccine hesitancy, with potential implications for the development of targeted strategies to support adult immunization programs.

Future research should prioritize under-represented regions, longitudinal designs, and standardized outcome measures, and should further explore the role of collective responsibility in HZ vaccination decisions.

## Figures and Tables

**Figure 1:**
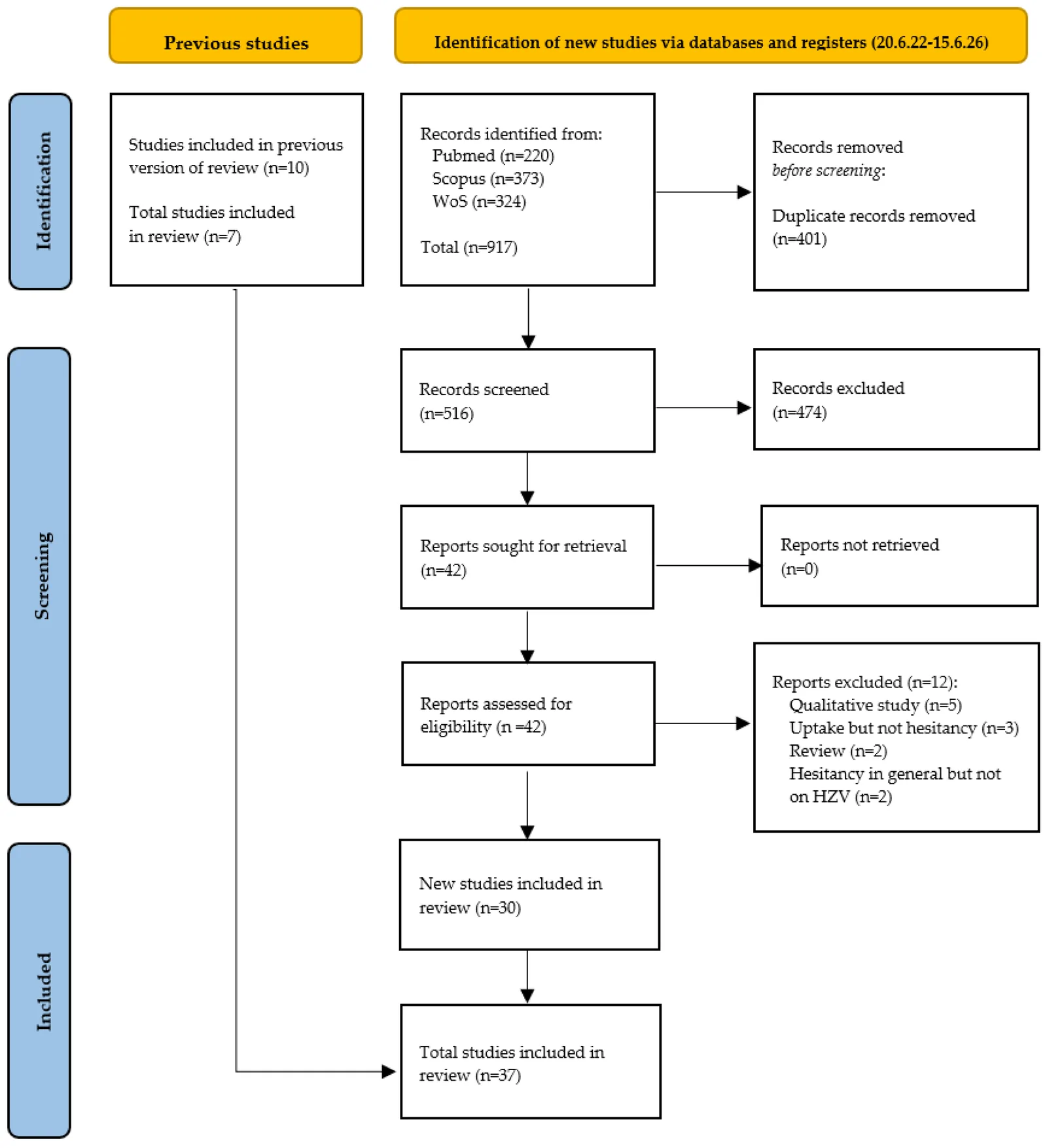
Flow diagram of the systematic literature search for updated systematic reviews, including searches of databases and registers only.

**Table 2:** Description of influencing factors in 5C model explored in the 37 included studies [32,33,34,35,36,37,38,39,40,41,42,43,44,45,46,47,48,49,50,51,52,53,54,55,56,57,58,59,60,61,62,63,64,65,66,67,68].

5C Model	Factor	Reference
Confidence	Trust in vaccine safety and effectiveness, healthcare professionals, and health authorities	[32,33,34,35,37,38,39,41,42,43,44,45,46,47,48,49,50,51,52,53,54,55,57,58,60,62,63,64,65,66]
Complacency	Low perceived risk of the disease, vaccination is considered unnecessary	[32,33,34,36,37,38,39,41,43,44,46,47,49,50,51,52,53,54,55,60,61,62,63,65,67]
Constraints	Structural barriers (availability, accessibility, affordability, and service delivery), psychological and self-regulatory obstacles	[32,33,34,37,38,39,41,43,45,46,47,49,50,51,53,55,61,62,64,68]
Calculation	Risk–benefit evaluation by actively engaging in information seeking	[33,34,37,39,40,41,42,43,44,45,47,49,50,52,53,54,55,57,58,60,63,65]
Collective Responsibility	Willingness to protect others through one’s own vaccination and contribute to community health	[37,39,49,64]

**Table 3:** Influencing factors explored in the 37 included studies using the 5C model [32,33,34,35,36,37,38,39,40,41,42,43,44,45,46,47,48,49,50,51,52,53,54,55,56,57,58,59,60,61,62,63,64,65,66,67,68].

First Author, Year, Country [Ref]	Endpoint (Hesitancy, Willingness, Acceptability)	Factors Associated with Endpoint					
		Socio-Demographic Determinants	5C Model				
			Confidence	Complacency	Constraints	Calculation	Collective Responsibility
Peng 2026 China [32]	Willingness *	Poor health status + (increases severity effect)	Response efficacy +	Perceived vulnerability to HZ +; Perceived severity of HZ = (but + for poor health group)	Response cost of HZV −; Self-efficacy +	-	-
Deng 2026 China [33]	Willingness/Hesitancy *	Gender F + (willingness) Age (≥70 y) − (hesitancy) Education =; Income =	Perceived prevention of complications by HZV +; HCP recommendation +; Awareness of local HZV policy +	Fear of HZ-related pain + (reduces complacency)	High out-of-pocket cost of HZV −	Awareness of HZV +	-
Lim 2026 Philippines [34]	Willingness *	Educational level =; Employment status =	Belief in efficacy of HZV +; Perceived health benefits of HZV +; HCP recommendation +	Personal risk perception of HZ +; Knowing someone with HZ =	Fear of adverse effects −; Out-of-pocket cost −	Knowledge of HZ/HZV =	-
Alrufayyiq 2026 Saudi Arabia [35]	Willingness (if recommended by HCP) *	Gender M +; Age =; Education =; Chronic diseases =	HCP recommendation +	-	-	-	-
Alolayan 2026 Saudi Arabia [36]	Willingness *	Dyslipidemia and cardiovascular issues −; Other diseases −	-	Knowing someone with shingles +; Knowing someone vaccinated with HZV +	-	-	-
Yang 2026 China [37]	Vaccination intention *	Monthly income +; Gender M + (in loss-frame)	Information credibility (IC) +; HCP recommendations +; Positive attitude +; Government vaccination policies +	HZ awareness +	Vaccine price acceptance +	eHealth literacy + (indirectly via IC/PC)	Family members’ influence +
Qu 2026 China [38]	Vaccination intention *	Age (≥60 y) −; Education =; Income =; Chronic diseases =	Perceived benefits of HZV +; Response efficacy of HZV +; Cues to action for HZV +	Perceived susceptibility to HZ +; Perceived severity of HZ +	Perceived barriers to HZV −; Self-efficacy +	-	-
Dai 2026 China [39]	Profile: willing but obstructed * (vs. passive acceptors)	Low income + (enhances cost barrier)	Lack of trust in vaccine safety/efficacy of HZV − (characterizes Distrustful profile)	Belief that HZ poses no personal threat − (characterizes Invulnerable profile)	High-cost barrier for HZV − (hinders Willing profile)	Over-analysis of pros and cons of HZV −	Willingness to protect others via HZV +
Zhang 2026 China [40]	Willingness *	Living alone =; Gender =; Age =; Chronic diseases =	-	-	-	Mistrust of vaccine benefit for HZV −; Worries about unforeseen future effects of HZV −	-
Liu 2026 China [41]	Willingness *	-	Perceived benefits of HZV + (indirect effect); Cues to action for HZV +	Perceived threat of HZ +	Perceived barriers to HZV −	eHealth literacy regarding HZV information +	-
Oku 2025 Japan [42]	Willingness *	Age (≥50 y) +; Gender M =	HCP recommendation +	-	-	Knowledge of HZV +	-
Hou 2025 China [43]	Hesitancy *	Gender F + (increases hesitancy); Income (2000–3000) − (reduces hesitancy); Health status + (increases hesitancy)	Trust in vaccine safety and information of HZV − (reduces hesitancy)	Overestimation of one’s health/Low perceived susceptibility to HZ + (increases hesitancy)	Perceived inconvenience of HZV + (increases hesitancy)	Awareness of HZ symptoms − (reduces hesitancy); Awareness of HZV (reduces hesitancy)	-
AlSaif 2025 Saudi Arabia [44]	Willingness *	Age =; Gender =; Education =; Chronic diseases =	Positive attitude score towards HZV +; HCP recommendation +	Family history of HZ +; Low perceived risk of HZ − (acts as a barrier)	-	Knowledge score on HZ/HZV +	-
Zhu 2025 China (Ningbo) [45]	Willingness *	Older age (≥60 y) −; High school or above +; Monthly income ≥US$685 +; Immune diseases − Vaccine-targeted diseases −; Self-rated health (fairly good/ average/poor) −	HCP recommendation +	-	Cost concerns −; Low income −	Knowledge score on HZ/HZV +	-
Gao 2025 China (Shanghai) [46]	Hesitancy *	Older age +; Urban residence +; Higher monthly income +	Response efficacy =	No history of HZ infection +	Low self-efficacy +; Extrinsic rewards +; Intrinsic rewards +; High response costs +	-	-
Liang 2025 Hong Kong [47]	Willingness *	Older age (70–74 y vs. 65–69 y) −; Secondary education (vs. primary or below) +; History of pneumococcal vaccination +; History of seasonal influenza vaccination +	Cue to action (HCP/family recommendation) − (self-financed only); Information exposure (government recommendation) +	Perceived susceptibility +; Information exposure (HZ risk in older adults and in COVID-19 survivors) +	Perceived self-efficacy +;	Perceived benefits +; Concern about side effects −	-
Motet 2025 Belgium [48]	Willingness *	-	Willingness to be vaccinated (any vaccine) +; Belief that vaccination is a good prevention tool +; General vaccine acceptance +	-	-	-	-
Wang 2025 China (Ningbo) [49]	Hesitancy	Older age +; Gender =; Urban/suburban residence +; Educational level =; History of underlying disease =; History of varicella =; Had the flu vaccine in 2022/2023 season =	Low confidence in vaccine safety/effectiveness +	High complacency +; Knowing someone who had HZ =	Lack of urban employee basic medical insurance +; Vaccine cost +; Limited healthcare resources +	Low vaccine-related knowledge +	Low collective responsibility +
Chang 2024 China [50]	Willingness	-	Absence of HCP recommendation −; Concerns about vaccine effectiveness +; Misconceptions about vaccines (e.g., treatment vs. prevention) +; Gaps in knowledge among both public and dermatologists +	Low perceived need for vaccination +; Underestimation of disease risk due to poor knowledge +	High vaccine cost +; Out-of-pocket payment +; Number of doses required +	Low knowledge about HZ/HZV +; Concerns about side effects +; Limited active information-seeking behavior +	-
Dyda 2024 Australia [51]	Hesitancy *	Older age (≥66 y) −; Gender F −; Urban residence +	Lower perceived importance of adult vaccination +; Lack of awareness of recommended vaccines +; HCP recommendation −	Low perceived risk of HZ +; Do not believe vaccination is necessary +; Belief that vaccination is more important for children +	Cost/out-of-pocket payment +; Low willingness to pay +; Being offered the vaccine −	-	-
Lasagna 2024 Italy [52]	Hesitancy, indirect	Older age (>75 years) −; Marital status (married/widowed) −	Lack of oncologist recommendation +; Lack of awareness of vaccination importance +	Vaccination not considered essential +; Low perceived importance of vaccination in cancer care +	-	Concerns about vaccine side effects +; Concerns about interference with cancer therapy +	-
Salussolia 2024 Italy [53]	Hesitancy *	Gender F +; Lower education +; Living in an island region +; Low worry about HZ +; Diabetes −; Living in rural areas −; Living in northeastern Italy −	Unaware of being target for vaccination +; Negative views of friends/family +; Friends/family against vaccination +	Low perceived risk of HZ +	Difficult access to vaccination +; Financial difficulties +	Perceive vaccine as unsafe +	-
Rubaian 2024 Saudi Arabia [54]	Willingness	-	Misconceptions about vaccine (e.g., treating disease) +; Preference for medications over vaccines +; Information mainly from family/friends +; Lack of HCP recommendation +	Belief that HZ is not serious +; Low prioritization of vaccination +	-	Concerns about side effects +; Low knowledge about HZ/HZV +; Information mainly from informal sources (family/friends) +	-
Zhang 2024 China [55]	Willingness *	Gender F −; Spouse with higher education +; Low frequency of influenza −; Chronic diseases −	HCP recommendation +	Low frequency of influenza (low perceived susceptibility) −; Awareness of link between chickenpox/HZ +	High vaccine cost +	Knowledge of HZ/HZV +; Having heard of HZV +	-
Grudziaz-Sekowska 2024 Poland [56]	Willingness *	Absence of children +; Moderate financial status +; Gender =; Age =; Education =; Married =; Place of residence =; Number of household members =; Occupational activity =; Chronic diseases diagnosed by a doctor =	-	-	-	-	-
Wang 2023 China [57]	Willingness *	Gender M −; Older age −; Education =; Annual net household income =; Underlying self-reported diseases =	Positive attitude +	-	-	Knowledge of HZ/HZV +	-
Di Giuseppe 2023 Italy [58]	Willingness *	Older age +; More than one chronic disease +; Received at least one recommended vaccination +; University degree =	Positive attitude on the usefulness of HZV +	-	-	Having heard of the availability of a HZV =; Feeling the need to receive additional information about HZV +	-
Maertzdorf 2023 Netherlands [59]	Willingness *	Older age +; Gender F −; Higher BMI +; More than two chronic diseases +; High educational level −; Employment =; Smoking habit =; Alcohol habit =; Being active =; Being hospitalized in the past 12 months =; Poor perceived health =	-	-	-	-	-
Al-Orini 2023 Saudi Arabia [60]	Acceptability *	Gender M +; education =; Occupation =; History of varicella vaccine uptake =	Believing that HZV is effective +	Knowing that immunocompromised individuals are at a higher risk of HZ +; Knowing about varicella disease =; Knowing about shingles =; History of varicella infection =; Knowing someone who had shingles =; History of shingles =	-	Knowledge of HZV =	-
	Acceptability if advised by HCP *	Gender M +; education =; Occupation =; History of varicella vaccine uptake +	-	Knowing about shingles =; Knowing someone who had shingles =; History of shingles =	-	Knowledge of HZV =	-
Jiang 2022 China [61]	Willingness *	High educational level −; Low monthly income +; Age =; Gender =; Profession =; Presence of underlying diseases =	-	Ever experiencing an episode of HZ =	Living in a rural area vs. urban area −	-	-
Al-Khalidi 2022 United Arab Emirates [62]	Willingness	Age =; Gender =; Nationality =; Educational attainment =; Employment status =; Occupation =; History of chronic diseases =	HCP recommendation +	History of chickenpox (yes vs. not sure) +	Insured =	-	-
Lu 2021 China [63]	Willingness to get HZV in self-payment scenario *	Younger age (59–59 vs. 60–69) +; Gender F +; Lower monthly income −; Lower educational level −	Vaccine hesitancy −; Knowing the target recipients of the HZV in China +; Knowing HZV schedule in China +	Knowing people in contact with those who have HZ are susceptible to contracting VZV +; Knowing the elderly are susceptible to contracting VZV +	-	Knowledge of HZ/HZV +	-
	Willingness to get HZV if covered by insurance *	Younger age (59–59 vs. 60–69) +; Gender F +; Lower monthly income −; Lower educational level −; More underlying chronic diseases +	Vaccine hesitancy −; Knowing the target recipients of the HZV in China +; Knowing the HZV schedule in China +	Knowing that people in contact with those who have HZ are susceptible to contracting VZV +; Knowing that the elderly are susceptible to contracting VZV +	-	Knowledge of HZ/HZV +	-
Nicholls 2021 UK [64]	Willingness *	Age =; Marital status =	Vaccine hesitancy −	-	Lower perceived social support −; Concerns about profiteering −	-	Lower sense of collective responsibility −
Del Signore 2020 France [65]	Acceptability *	Gender M +; Younger age +; Patients enrolled from private laboratories (vs. from hospital department) +; Educational level =; Chronic diseases =; Marital status (in a relationship/married vs. single) +	Who would agree to be vaccinated against any infectious diseases +; Who thinks that vaccination is a good prevention tool +	Who thinks that HZ is always severe +; Who thinks that HZ causes pain +; Personal history of HZ =; Treatment for HZ =; Personal history of PHN =; Treatment for PHN =; Impairment in activities of daily living due to PHN =; Who knows someone with a history of HZ =	-	Knowledge of HZV =	-
Baalbaki 2019 USA [66]	Acceptability	Older age +	HCP recommendation +	-	-	-	-
Yang 2015 South Korea [67]	Willingness *	Younger age +; Gender =; Education (high vs. low) +; Higher monthly household income +; Self-reported rate of receiving the annual seasonal flu vaccine =	-	Personal history of HZ =; Knowing someone with HZ =	-	-	-
Lu 2009 USA [68]	Willingness *	Age =; Gender =; Ethnicity =; Higher income +; Education =; Married status =	-	-	Medical insurance status =	-	-

* Multivariate logistic regression analysis, +: Positive association with endpoint, −: Negative association with endpoint, =: No significant association with endpoint, -: Not reported, HCP: Healthcare Professional, HZ: Herpes Zoster, HZV: Herpes Zoster Vaccine, VZV: Varicella Zoster Virus.

## Data Availability

No new data were created or analyzed in this study.

## Supplementary Materials

The following supporting information can be downloaded at: https://www.mdpi.com/article/10.3390/vaccines14100840/s1, Table S1: Search strategy; Table S2: New Castle Ottawa Scale of 37 included studies [32,33,34,35,36,37,38,39,40,41,42,43,44,45,46,47,48,49,50,51,52,53,54,55,56,57,58,59,60,61,62,63,64,65,66,67,68]; Table S3: Prisma 2020 Checklist.
